# Heat Dissipation in Epoxy/Amine-Based Gradient Composites with Alumina Particles: A Critical Evaluation of Thermal Conductivity Measurements

**DOI:** 10.3390/polym10101131

**Published:** 2018-10-11

**Authors:** Matthias Morak, Philipp Marx, Mario Gschwandl, Peter Filipp Fuchs, Martin Pfost, Frank Wiesbrock

**Affiliations:** 1 Polymer Competence Center Leoben GmbH, Roseggerstrasse 12, 8700 Leoben, Austria; matthias.morak@pccl.at (M.M.); philipp.marx@pccl.at (P.M.); mario.gschwandl@pccl.at (M.G.); peterfilipp.fuchs@pccl.at (P.F.F.); 2 Chair of Mechanics, Montanuniversitaet Leoben, Franz-Josef-Strasse 18, 8700 Leoben, Austria; 3 Chair of Chemistry of Polymeric Materials, Montanuniversitaet Leoben, Otto Gloeckel-Straße 2, 8700 Leoben, Austria; 4 Chair of Energy Conversion, TU Dortmund University, Emil-Figge-Strasse 50, 44227 Dortmund, Germany; martin.pfost@tu-dortmund.de; 5 Institute for Chemistry and Technology of Materials, Graz University of Technology, NAWI Graz, Stremayrgasse 9, 8010 Graz, Austria

**Keywords:** epoxy/amine resin, alumina particles, thermal conductivity, thermal management, gradient composite

## Abstract

For the design of the next generation of microelectronic packages, thermal management is one of the key aspects and must be met by the development of polymers with enhanced thermal conductivity. While all polymer classes show a very low thermal conductivity, this shortcoming can be compensated for by the addition of fillers, yielding polymer-based composite materials with high thermal conductivity. The inorganic fillers, however, are often available only in submicron- and micron-scaled dimensions and, consequently, can sediment during the curing reaction of the polymer matrix. In this study, an epoxy/amine resin was filled with nano- and submicron-scaled alumina particles, yielding a gradient composite. It was found that the thermal conductivity according to laser flash analysis of a sliced specimen ranged from 0.25 to 0.45 W·m^−1^·K^−1^ at room temperature. If the thermal conductivity of an uncut specimen was measured with a guarded heat flow meter, the ‘averaged’ thermal conductivity was measured to be only 0.25 W·m^−1^·K^−1^. Finite element analysis revealed that the heat dissipation through a gradient composite was of intermediate speed in comparison with homogeneous composites exhibiting a non-gradient thermal conductivity of 0.25 and 0.45 W·m^−1^·K^−1^.

## 1. Introduction

Thermal management is one of the key aspects in the design of reliable microelectronic packages [1,2] as well as high-voltage machinery [3,4]. Geometry and materials have to be defined in a way that the packages, insulations, multi-layer assemblies, etc. can withstand the application-specific external or internal temperature loads. In microelectronics, the most critical load often originates from an internally generated heat by active components such as a power metal-oxide-semiconductor field-effect transistor (MOSFET) [5,6,7,8]. This type of silicon chip can produce high temperatures in short times, which results in pronounced temperature gradients and high thermo-mechanical strains [9]. Due to the ongoing demand for integrated functions and miniaturization, geometric changes of the design are subject to limitations, which add additional importance to increasing the thermal conductivity of the materials used [10].

Polymers are commonly used materials in power packages. Despite their broad versatility in material properties and functionality, all polymer classes show one common physico-chemical characteristic, namely, a very low thermal conductivity commonly in the range of 0.1 to 0.2 W·m^−1^·K^−1^ [4]. Very few polymers with dedicated structural motifs show a higher thermal conductivity of 0.3 W·m^−1^·K^−1^ [11]. This characteristic can be overcome by the addition of fillers with high thermal conductivity [4,12,13,14]. Due to the low heights of films in power packages or insulation layers down to the μm range, such (inorganic) fillers are preferentially used in nano- and/or submicron-scaled sizes. Aiming at high thermal conductivity of a composite material, highly priced fillers with high thermal conductivity such as nanodiamonds (λ = 2200 W·m^−1^·K^−1^) [15], hexagonal boron nitride (λ = 390 W·m^−1^·K^−1^) [16], and/or aluminum nitride (λ = 300 W·m^−1^·K^−1^) [17] may be added; reasonably priced alternatives are comprised of silica (λ = 0.7 W·m^−1^·K^−1^) and alumina (λ = 23 W·m^−1^·K^−1^) [18].

In cases of some inorganic fillers, nano-scaled particles are not available in quantities relevant for industrial production and/or at reasonable prices; this is particularly true for nanodiamonds and hexagonal boron nitride due to, among other things, their hardness. It must be taken into account that particles with diameters above the nano-scale may sediment in a composite material during the curing reaction, yielding a gradient composite with varying composition along the height scale (e.g., increasing content of inorganic fillers from top to bottom) and, consequently, an analogously varying thermal conductivity. The phenomenon of sedimentation eventually occurs to even higher extent due to the agglomeration of (non-functionalized) particles, despite their initial homogeneous dispersion in a polymer matrix [14].

Correspondingly, this study aimed at investigating the effect of particle sedimentation in an epoxy/amine resin and the consequences on the thermal conductivity of and the thermal dissipation through such a composite gradient. In order to trigger sedimentation of the particles, a mixture of nano- and submicron-scaled alumina particles was used. The thermal conductivity was comparatively quantified by laser flash analysis and a guarded heat flow meter. The experimental study was complemented by modelling efforts and finite element analysis in order to detail the thermal properties of gradient composites. With the help of numerical simulations based on thermal finite element models, the thermal properties of various types of materials, including the gradient composites detailed in this study, can be calculated in straightforward fashion, as this type of simulation models predominantly considers the thermal flux through the material. As such, it inherently provides the opportunity to calculate the temperature field without consideration of stresses, deformations, or electrical fields.

## 2. Materials and Methods

### 2.1. Materials

**Figure 1:**
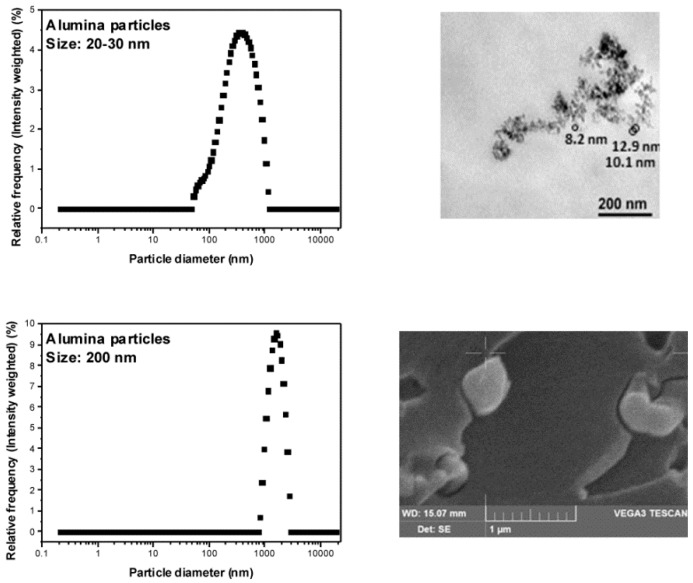
Dynamic light scattering (DLS) measurements of particles in water (**left**) as well as TEM/SEM images of the particles embedded in a polymer matrix (**right**), referring to the alumina nanoparticles (**top**) and the alumina submicron particles (**bottom**). The ranges of diameters of 20–30 and 200 nm, respectively, which were provided by the suppliers, could be verified by the TEM/SEM images; the DLS measurements revealed aggregation of the particles.

Bisphenol A diglycidyl ether (DGEBA) and diethylenetriamine (DETA) were purchased from Sigma Aldrich (Vienna, Austria). The Al_2_O_3_ nanoparticles (20–30 nm) and Al_2_O_3_ submicron-particles (200 nm) were bought from ABCR (Karlsruhe, Germany). The range of diameters of the particles provided by the suppliers was verified by transmission electron microscopy (TEM and scanning electron microscopy (SEM) measurements of polymer films containing one type of alumina particles; dynamic light scattering (DLS) measurements failed to reproduce the diameters of the individual particles due to aggregation of the non-functionalized alumina particles (Figure 1). All chemicals were used as received.

### 2.2. Instrumentation

The thermal diffusivity was measured on a LFA 467 Hyperflash system (Netzsch, Selb, Germany) at temperatures of 20, 40, 60, 80, 100, 120, and 140 °C. The samples were coated with a thin graphite layer before the measurement in order to avoid reflection. The measurements of the specific heat capacity were performed in duplicate on DSC 6000 (PerkinElmer, Vienna, Austria) using sapphire as reference material. The samples were heated up twice from −10 to 150 °C with a heating rate of 10 K·min^−1^. The density was determined with a hydrostatic balance XS205 Dual Range (Mettler-Toledo GmbH, Vienna, Austria) by weighing samples in air and water at 25 °C. The thermal conductivity of the uncut gradient composites was measured on a DTC-300 thermal conductivity meter (TA Instruments, New Castle, DE, USA) according to the norm ASTM E1530 at temperatures of 20, 40, 60, 80, 100, 120, and 140 °C, using specimens with diameters of 50 mm and heights of 10 mm. For the dispersion of the nano- and submicroparticles in the resins, the Dissolver Dispermat AE 03 (VMA-Getzmann GmbH, Reichshof, Germany) was used (20 min, 5000 rpm). Particle sizes were measured in aqueous media (5 mg/20 g) by dynamic light scattering using a Litesizer 500 (Anton Paar, Austria). TEM images were recorded on a FEI Tecnai 12 transmission electron microscope. SEM-EDX measurements were performed using a Tescan Vega 3 scanning electron microscope with an energy dispersive X-ray spectrometer (EDX Oxford Instruments INKAx-act, (High Wycombe, UK)) attached. Electron energy levels were set to 20 kV.

### 2.3. Preparation of the Gradient Epoxy-Amine-Based Composite

For the preparation of the test specimens containing 20 wt % of Al_2_O_3_ particles, 24.39 g of Al_2_O_3_ nanoparticles and 24.39 g of Al_2_O_3_ submicroparticles were added to 174 g of DGEBA. In order to achieve a homogenous dispersion, the mixture was stirred with a high-shear mixer at r.t. Subsequently, the particle-DGEBA dispersion was mixed with DETA in a mass ratio of 10.56:1 (molar ratio of DGEBA:DETA = 2.5:1) and stirred for 5 min at r.t. For the removal of air bubbles, the mixture was sonicated for 5 min, poured into a mold with the targeted geometry, cured at r.t. for 4 h, and post-cured at 120 °C for 1 h. For the LFA measurements, a specimen with a diameter of 25 mm and a height of 10 mm was cut into layers with a thickness of approximately 800 μm using a diamond-equipped microtome. For the measurement of the “averaged” thermal conductivity of the bulk material, a specimen with a diameter of 50 mm and a height of 10 mm was produced.

### 2.4. Simulation Model

In order to evaluate the effect of a material with gradual thermal conductivity distribution, a simulation model using an uncoupled heat transfer analysis was employed. The term “uncoupled” summarizes that the temperature field was calculated without consideration of the stresses, respectively deformations, or any electrical fields; the model nonetheless can include conduction, boundary convection, and boundary radiation. Since in the present case the material properties are temperature-dependent, the heat transfer analysis is nonlinear. In order to consider the internal energy within the calculation, a transient analysis was carried out, in which the time integration is performed according to the backward Euler scheme [19]. The basic equation for an uncoupled heat transfer analysis is the energy balance equation (Equation (1)) [20]

(1) $$\int_{\Omega }\rho \dot{U}dx=\int_{\partial \Omega }qds_{x}+\int_{\Omega }rdx$$

in which Ω is the volume of a solid material, ∂Ω the surface of the volume, ρ the density, $\dot{U}$ the rate of the internal energy and where U=U(θ) with the temperature θ, q the heat flux, and r the internal heat.

The internal energy depends on the temperature only, which is usually defined in terms of a specific heat (Equation (2))

(2) $$c\left(\theta \right)=\frac{dU}{d\theta }$$

The equation for the thermal equilibrium can be derived from the energy balance equation (Equation (1)) by means of the specific heat and the term of heat conduction governed by Fourier’s law (Equations (3) and (4))

(3) $$f=-k\frac{d\theta }{dx}\forall \;x\in \Omega$$

(4) $$f_{\partial \Omega }=-n\cdot k\cdot \frac{d\theta }{dx}\forall \;x\in \partial \Omega$$

The overall energy balance equation (Equation (5)) [21] is obtained as

(5) $$\int_{\Omega }\delta \theta \left[\rho c\frac{\partial \theta }{\partial t}-\frac{\partial }{\partial x}\cdot \left(k\cdot \frac{\partial \theta }{\partial x}\right)-q\right]dx+\int_{\partial \Omega }\delta \theta \left[n\cdot k\cdot \frac{\partial \theta }{\partial x}-q_{s}\right]ds_{x}=0$$

in which δΩ is the variational field satisfying the essential boundary conditions, ρ(θ) the temperature-dependent density, c(θ) the temperature-dependent specific heat, k(θ)=k(θ)I the isotropic temperature-dependent thermal conductivity, q the heat added from arbitrary heat sources, and $q_{s}=h\left(\theta -\theta_{0}\right)$ the surface convection with the film coefficient h.

**Figure 2:**
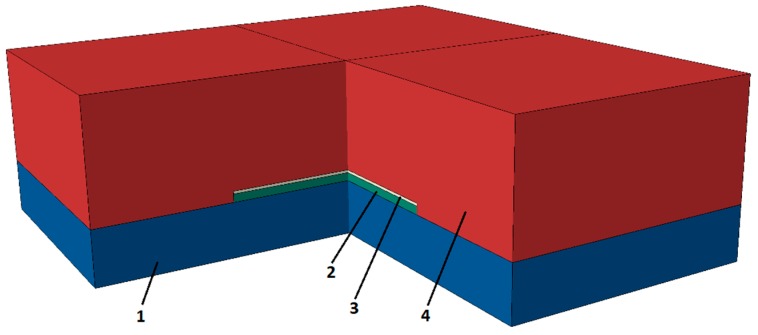
Profile of the simulation model. Part 1 and part 3 are copper layers, part 2 is a silicon layer, and part 4 is the epoxy-amine-based composite, which was divided in eight layers.

The simulation model itself consists of different parts considering the individual dimensions and material parameters (Figure 2, Table 1 and Table 2). Additionally, the measured data of the epoxy resin (Figures 6 and 7) were assigned to the corresponding layers of the model.

**Figure 3:**
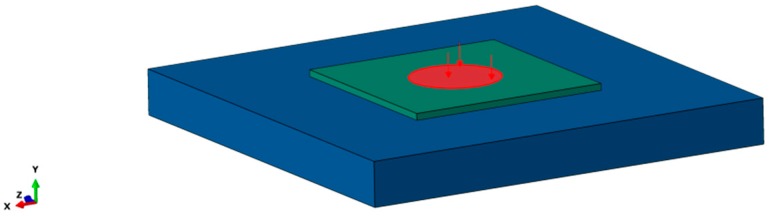
Area of the simulation model, in which the surface heat flux was applied (cp. Figure 2).

In the field of microelectronics, a wide power range from mW up to GW exists. Hence, at the beginning of the analysis, several values for the heat flux were tested, aiming to find a suited value enabling to reach the temperature values that can occur during the power thermal cycle (PTC) test [22]. In final consequence, a surface heat flux of $75\frac{W}{\mathrm{area}}$ was applied in a region with a radius of 1 mm on top of the silicon layer (Figure 3). This results in a heat flux of approx. 23.87 W·mm^−2^.

The entire model system was meshed with 8-noded linear heat transfer brick elements (DC3D8), respectively, with 4-noded linear heat transfer tetrahedral elements (DC3D4), whereas the total amount of elements of the epoxy layer was 112,664 tetrahedral elements. In order to demonstrate the effects of the gradual heat conductive distribution, three simulations with different material data of the epoxy-amine-based composite were performed: firstly, a simulation with the layer-wise measured epoxy data (Figures 6 and 7), secondly, a simulation with the data gained from the guarded heat flow meter (GHFM) measurements, and, finally, an idealized simulation, in which it was assumed that each of the eight epoxy layers entails the highest thermal conductivity measured (bottom layer 8 in Figures 6 and 7). Since the calibration of the film coefficient was impossible, several film coefficients were evaluated. The overall behavior remains the same, independent of the film coefficient, only the absolute temperature values are shifted and, accordingly, the temperature difference between the simulation results is more or less pronounced in dependence of the film coefficient. Boundary convection was taken into account by defining a film coefficient of $10\frac{W}{m^{2}K}$ from literature [23].

**Figure 4:**
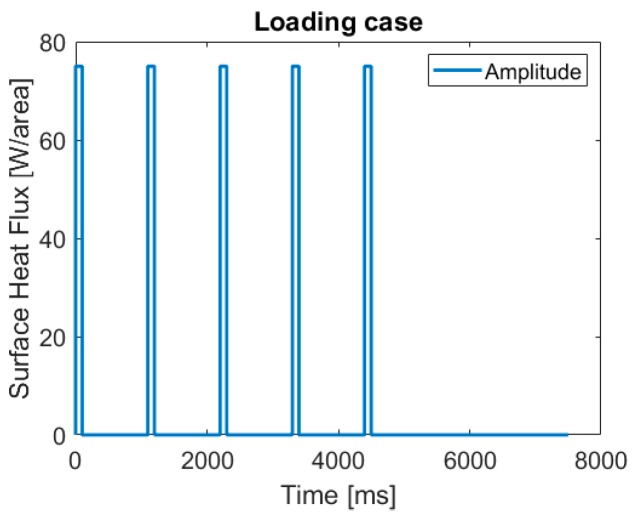
Thermal loading and unloading steps.

As initial temperature condition, r.t. of 23 °C was considered. A cyclic thermal loading and unloading was applied (Figure 4). In total, five loading cycles with a surface heat flux of $75\frac{W}{\mathrm{area}}$ of 0.1 s, interrupted by five unloading cycles of 1 s (after the first four loadings), respectively 3 s (after the fifth loading), were applied, yielding a total simulation time of 7.5 s. The thermal loading cycle with the abovementioned parameters was chosen in alignment to the power thermal cycle (PTC) test, which is one type of conventional test method in the field of microelectronics [22,24].

## 3. Results

### 3.1. Concept and Preparation of the Gradient Composites

**Figure 5:**
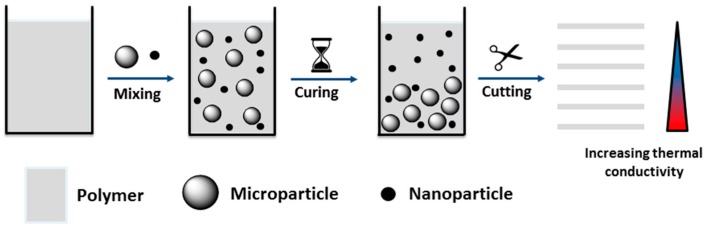
Schematic overview of the concept and the preparation of gradient composites containing a mixture of nano- and submicron-scaled particles.

In this study, the thermal conductivity of a gradient composite based on an epoxy-amine resin composed of DGEBA and DETA was investigated. For the preparation of the test specimens, 10 wt % of alumina submicroparticles (diameter of 200 nm) and 10 wt % of alumina nanoparticles (diameter of 20–30 nm) were dispersed homogenously in the epoxy resin using a high-shear mixer. Subsequently, the resin was cured at r.t. for 4 h. Due to their higher weight, the submicroparticles were expected to sediment during the curing reaction (Figure 5), while the smaller nanoparticles were expected to remain dispersed throughout the resin. Due to the sedimentation, a gradient of the particle distribution was expected to occur and, correspondingly, a gradient of the thermal conductivity over the height of the specimen. In order to quantify the gradient of the thermal conductivity, the cured composite specimens with heights of 10 mm were cut into layers with a thickness of approximately 800 μm using a microtome. The thermal conductivity of each individual layer was measured with a laser flash analysis system.

### 3.2. Measurement of the Thermal Conductivity

The thermal conductivity is one key thermo-physical material parameter, quantifying the heat transfer through a material. Several measurement techniques are available for the determination of the thermal conductivity. In case of thin specimens, laser flash analysis (LFA) is considered the most suitable approach for the determination of thermal conductivity, which is a contactless, non-destructive, and transient measurement approach. A very short energy pulse (e.g., laser or light pulse) is applied to heat the front surface of a plane-parallel specimen, while, on the rear surface, an infrared detector is used to measure the temperature increase. Assuming a fully adiabatic system, the thermal diffusivity *a*(*T*) of the sample can be calculated using the half-life period *t*_1/2_ of the temperature increase and the sample height d (Equation (6)) [25]

(6) $$a\left(T\right)=0.1388\frac{d²}{t_{1/2}}$$

The LFA is an indirect measurement method for the thermal conductivity *λ*(*T*) as it measures the thermal diffusivity *a*(*T*). Nonetheless, using *a*(*T*), the density *ρ*(*T*) and the specific heat capacity *c_p_*(*T*), *λ*(*T*) can be calculated (Equation (7))

(7) $$\lambda \left(T\right)=a\left(T\right)\rho \left(T\right)c_{p}\left(T\right).$$

Consequently, additional measurements are necessary, for differential scanning calorimetry (DSC) for the specific heat capacity measurement as well as a hydrostatic balance for the density measurement. Note that, the density is only characterized at room temperature, due to the negligible change over temperature.

**Figure 6:**
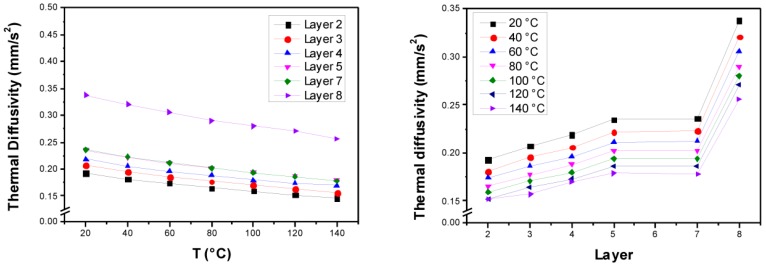
(**Left**) Thermal diffusivity *a*(*T*) of the different layers according to laser flash analysis (LFA) measurements. (**Right**) Gradient of the thermal diffusivity along the different layers.

As mentioned above, the composites were cut into evenly dimensioned layers, all of which were measured, unless their surfaces were too rough for uncorrupted LFA measurements (layers number 1 and 6). All layers show densities in the range of 1.33 to 1.38 g·cm^−3^ (Table 3). The LFA measurements of the layers show a clearly visible gradient of the thermal diffusivity *a*(*T*) over the different layers ranging from 0.20 to 0.35 mm^2^·s^−1^ at r.t. (Figure 6). The bottom layer (layer number 8 at position *h* = 8.448 mm) shows a pronouncedly higher thermal diffusivity in comparison with the other layers. Hence, it may be assumed that most of the (submicron-scaled) particles sedimented in the composite material during the curing reaction. The results of the consecutive layers (layer numbers 7 to 2) as well show a gradient of the thermal diffusivity.

**Figure 7:**
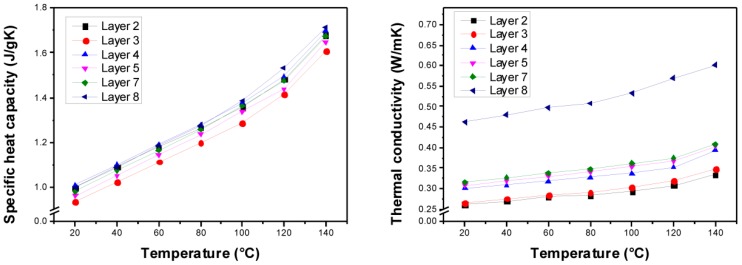
(**Left**) Specific heat capacity measurements for the different layers using differential scanning calorimetry (DSC). (**Right**) Calculated thermal conductivity of the different layers based on the measurements of the thermal diffusivity, the density, and the specific heat capacity.

The measurement of the specific heat capacity *c_p_*(*T*) by DSC yielded no gradient behavior. In the temperature range from 20 to 140 °C, *c_p_*(*T*) varied from approx. 1.0 to 1.6 J·g^−1^·K^−1^ (Figure 7 left). The non-gradient behavior can be referred to the similar specific heat capacities of the filler Al_2_O_3_ (*c_p_*(293 K) = 0.8 J·g^−1^·K^−1^ [26]) and the epoxy/amine resin (*c_p_*(293 K) = 1.2 J·g^−1^·K^−1^ [27]). As the density and the specific heat capacity vary to a very small extent, only within an individual layer at a given temperature, the thermal conductivity is quasi-proportional to the thermal diffusivity (Equation (7)), and the thermal conductivity shows similar trends like the thermal diffusivity (Figure 7 right). The thermal conductivity of the bottom layer (layer number 8) is significantly increased, likely due to the assumed filler agglomeration in that layer, while the consecutive layers display a clear gradient to the topmost layer (layer number 2). In summary, the thermal conductivity at r.t. ranged from 0.25 to 0.45 W·m^−1^·K^−1^; unfilled epoxy-amine resins exhibit a thermal conductivity of approx. 0.2 W·m^−1^·K^−1^ [14].

For the determination of the averaged thermal conductivity of such gradient composites, also GHFM measurements of a non-sliced specimen had been carried out. This method is well-established and commonly used for the thermal conductivity measurement of polymers [28,29,30]. The GHFM is a stationary measurement method, whereby the specimen is placed between a hot and cold plate. A heat flux transducer is used, which determines the steady heat flow *Q* through the specimen. Using Fourier’s law, the thermal conductivity can be calculated (Equation (8))

(8) $$\lambda \left(T\right)=\frac{Qd}{\Delta TA}$$

in which Δ*T* is the temperature difference between the hot and cold plate, *A* is the area of the specimen’s surface that is in contact with the heating plates, and *d* is the thickness of the sample. 

**Figure 8:**
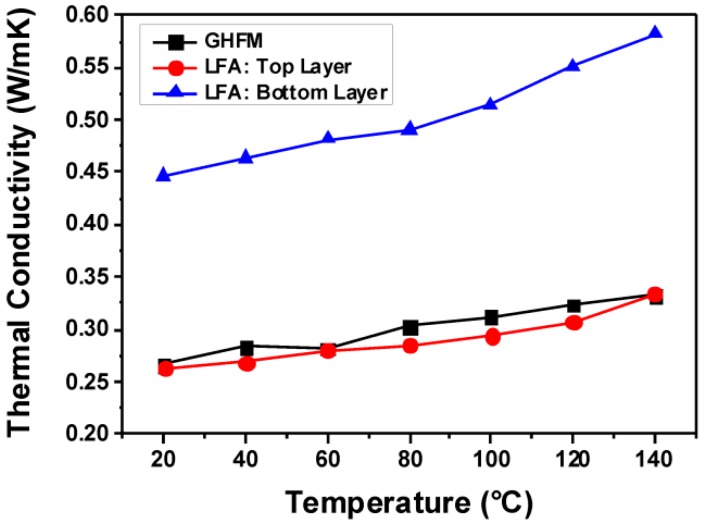
Comparison of the guarded heat flow meter (GHFM) (representing a homogenized bulk material) and the LFA measurements (representing the respective layers of a gradient composite numbered 2 and 8) of the epoxy resin-based composite.

The GHFM measurements reveal a thermal conductivity of the bulk material of 0.25 W·m^−1^·K^−1^ at r.t. (Figure 8). Apparently, over the whole range of temperatures from 20 to 140 °C, the thermal conductivity of the bulk material reproduces the thermal conductivity observed in the top layer (layer number 2) of the cut specimen (see hereinabove), which is the layer with the lowest thermal conductivity. This measurement has been performed in triplicate. Tt must be argued that the overall thermal conductivity of the bulk material according to GHFM measurementscorresponds to the lowest thermal conductivity of an individual layer of the gradient material measured by LFA. Hence, in GHFM measurements, the overall thermal conductivity of an inhomogeneous material is limited by the lowest thermal conductivity present in the (gradient) material. The heat dissipation from ‘hot spots’ in vicinity to such gradient materials, consequently, cannot be discussed in sufficient detail with respect to (only) the ‘averaged’ thermal conductivity. Instead, the gradient behavior of the thermal conductivity must also be considered. For a more elaborate discussion of the thermal dissipation behavior, this behavior was modelled and simulated in this study.

### 3.3. Modelling & Simulation of the Thermal Dissipation Behavior

**Figure 9:**
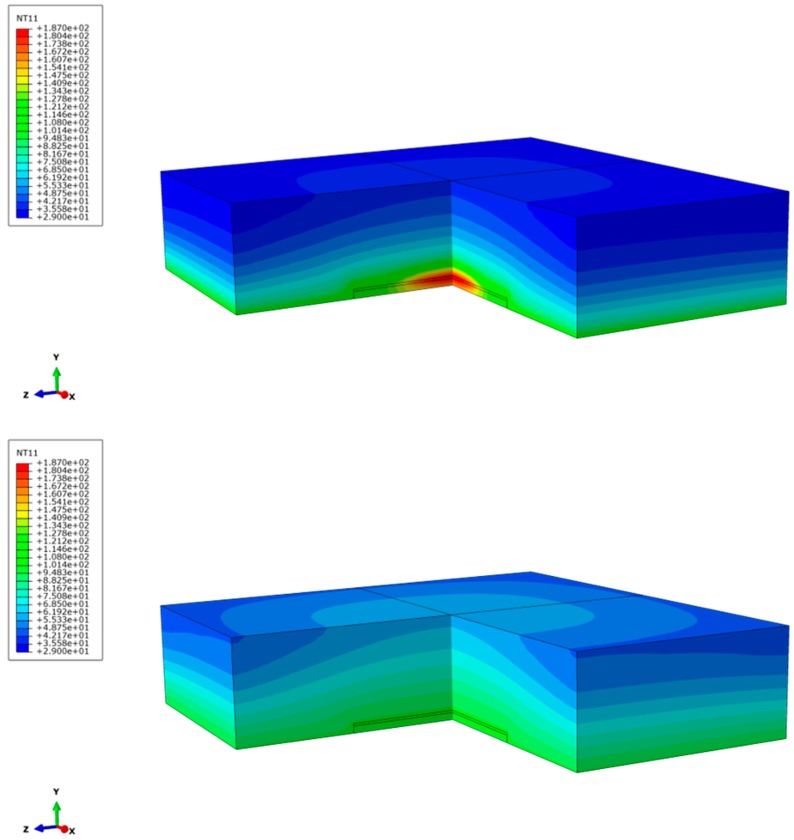
(**Top**) Resulting temperature field immediately after the last loading step for the layer-wise measured epoxy data. (**Bottom**) Resulting temperature field at the end of the last unloading step for the layer-wise measured epoxy data.

For the simulation of a gradual thermal conductivity distribution, a model with an uncoupled heat transfer analysis, including conduction, boundary convection, and boundary radiation, was employed. A multi-layer model composed of copper, silicon, and an epoxy-based composite was employed (Figure 2). For the epoxy material, eight layers were considered, the material parameters of which were taken from the measurements described above. A cyclic thermal loading and unloading with five loading cycles of 0.1 s each was applied (Figure 4). The heat was applied in a region with a radius of 1 mm on top of the silicon layer; the first four loading cycles were interrupted by unloading cycles of 1 s, while the last loading cycle was ended with an unloading cycle of 3 s. The temperature fields immediately after the last loading cycle (Figure 9 top) and 3 s after the last loading cycle (Figure 9 bottom) show a gradual dissipation of the heat through the epoxy-based material. Expectedly, the maximum temperature is reached in the region where the heat was applied and decreases throughout the epoxy. It can be clearly seen that the heat is transferred to the top of the epoxy structure in gradual fashion, which evolves over time.

**Figure 10:**
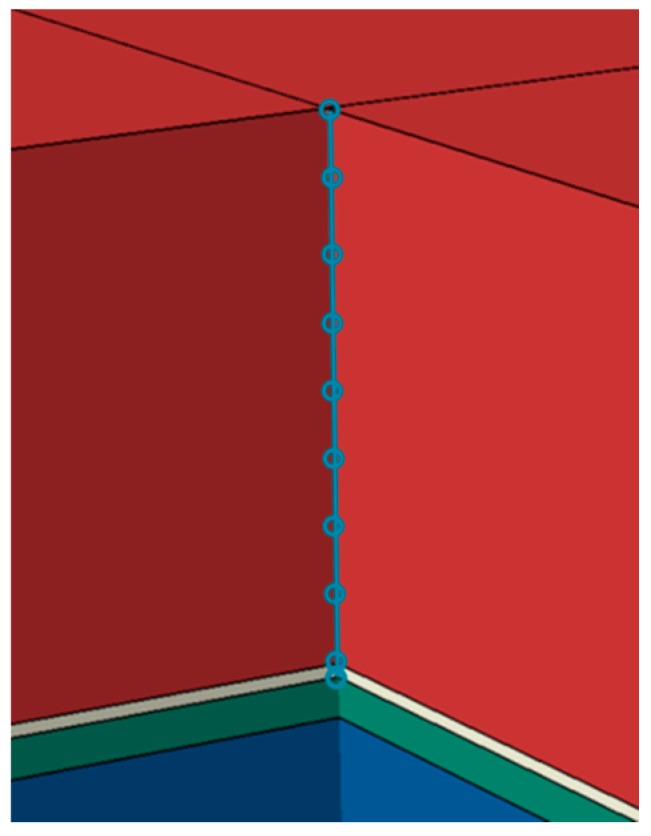
Evaluation points on top of the silicon layer, on top of the copper layer, and on top of each of the eight epoxy layers.

For the evaluation of the (time-resolved) heat transfer, a path along the copper/silicon/epoxy materials with several examination points was defined, comprising the top of the silicon layer, the top of the copper layer, and the top of each of the eight epoxy layers (Figure 10). For the calculations, three sets of material parameters for the epoxy layer were considered, namely (i) “averaged” thermal conductivity for a non-gradient composite material (reproducing the GHFM measurements); (ii) gradient thermal conductivity according to the LFA measurements of individual layers of a gradient composite material; and (iii) high thermal conductivity for a non-gradient composite material (reproducing the LFA measurement of the bottom layer number 8).

**Figure 11:**
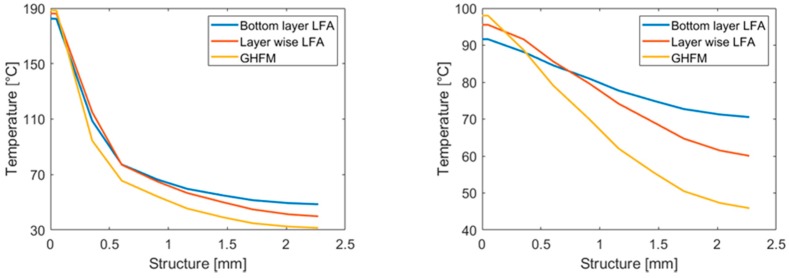
(**Left**) Temperature distribution immediately after the last loading cycle throughout the defined path (Figure 10). (**Right**) Temperature distribution at the end of the last unloading cycle throughout the defined path (Figure 10).

From the temperature distribution along this path immediately after the last loading cycle (Figure 11 left), it is perceptible that the temperature on the silicon layer is, as expected, at its maximum with the “averaged” GHFM-measured data, because of the low thermal conductivity considered for the bulk material. Correspondingly, the lowest temperature is obtained on the top-boundary of the epoxy structure. Considering a non-gradient material with high thermal conductivity, the temperature is highest on the top-boundary of the epoxy structure and lowest on the silicon layer. The heat convection of the simulation using the layer-wise measured data of the epoxy-amine-based composite is located between the results of the simulation with the GHFM-measured data and the results with the idealized epoxy data. Notably, 3 s after the thermal loading (Figure 11 right), these effects become even more pronounced, revealing a temperature difference of approximately 20 K on the top-boundary of the epoxy structure, between the layer-wise model, according to LFA measurements and the model with ‘averaged’ low thermal conductivity, according to GHFM measurements.

**Figure 12:**
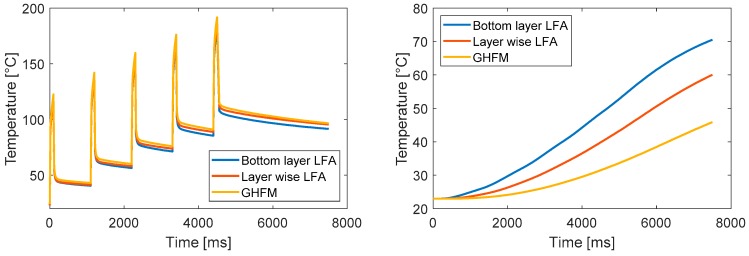
(**Left**) Temperature distribution over the total simulation time, measured at the top of the silicon layer. (**Right**) Temperature distribution over the total simulation time measured at the uppermost epoxy layer.

The comparison of the temperature evolution over time for all layers reveals that the temperature difference between the three simulation results increases for each layer (Figure 12). Whilst at the top of the silicon layer, cyclic temperature lifts are observed (Figure 12 left) and continuous heating occurs at the top of the epoxy structure (Figure 12 right). Especially at the silicone-epoxy interface, an enhancement of the thermal conductivity is noticeable after several temperature lifts. 

## 4. Summary, Discussion and Conclusions

An epoxy/amine-based gradient composite was prepared by the addition of nano- and submicron-scaled alumina particles by expanding the curing time of the polymer matrix to the range of a few hours. The thermal conductivity within this gradient composite ranged from 0.25 to 0.45 W·m^−1^·K^−1^ at r.t., hence by a factor of almost 2. The range of thermal conductivities could be measured within this study only by performing laser flash analysis on individual layers of a dedicatedly cut specimen. If the thermal conductivity of an uncut specimen was measured with a guarded heat flow meter, the ‘averaged’ thermal conductivity was measured to be 0.25 W·m^−1^·K^−1^, reproducing in good approximation the lowest value of thermal conductivity according to laser flash analysis. The fact that these two types of measurement techniques revealed pronouncedly different findings will be subject of further studies. Complementary finite element analysis of a multi-layer assembly comprising layers of copper, silicon, and the epoxy/amine/alumina composite revealed that the heat dissipation through a gradient composite was of intermediate speed in comparison with materials exhibiting a non-gradient thermal conductivity of 0.25 and 0.45 W·m^−1^·K^−1^, respectively, if the layers with the highest thermal conductivities were adjacent to the heat source. These findings of the finite element analysis supported the experimental data of the laser flash analysis. 

It may be concluded from this study that gradient composites are formed (autonomously) within the range of a few hours if non-nanoscaled fillers are added to a polymer matrix, e.g., for the enhancement of the thermal conductivity. This phenomenon must be taken into account in particular if only submicron or micron-scaled fillers are (commercially) available. Correspondingly, the thermal conductivity varies along (at least) one dimension of such a gradient composite. Such gradient composites nonetheless enable faster heat dissipation compared to a homogeneous composite material with a uniform thermal conductivity identical to the lowest one of the gradient composite, despite eventually identical thermal conductivity according to guarded heat flow Meter measurements.

## Figures and Tables

**Table 1:** Parts and layer dimensions of the model.

Layer	Length (mm)	Width (mm)	Height (mm)
Copper (layer 1)	9.5	9.5	1
Silicon	4.5	4.5	0.15
Copper (layer 2)	4.5	4.5	0.05
Epoxy	9.5	9.5	2.2
Epoxy layers	0.3125	0.3125	0.25

**Table 2:** Material parameters according to literature data.

Material	Temperature (°C)	$\mathrm{Thermal}\;\mathrm{Conductivity}\;\left(\frac{kW}{mK}\right)$	$\mathrm{Density}\;\left(\frac{g}{mm^{3}}\right)$	$\mathrm{Specific}\;\mathrm{Heat}\;\left(\frac{J}{kgK}\right)$
Copper	25	4.0 × 10^−1^	8.93 × 10^−3^	3.85 × 10^2^
Silicon	25	1.670 × 10^−1^	2.329 × 10^−3^	5.769 × 10^2^
	400	5.257 × 10^−2^	2.329 × 10^−3^	8.741 × 10^2^

**Table 3:** Position and density of the individual layers.

Height Position (mm)	Layer (#)	Density *ρ* (g·cm^−3^)
1.592	2	1.364
2.767	3	1.376
3.856	4	1.364
4.961	5	1.367
7.177	7	1.368
8.448	8	1.372
